# Metabolic Regulation of Epigenetic Modifications and Cell Differentiation in Cancer

**DOI:** 10.3390/cancers12123788

**Published:** 2020-12-16

**Authors:** Pasquale Saggese, Assunta Sellitto, Cesar A. Martinez, Giorgio Giurato, Giovanni Nassa, Francesca Rizzo, Roberta Tarallo, Claudio Scafoglio

**Affiliations:** 1 Division of Pulmonary and Critical Care Medicine, David Geffen School of Medicine, University of California Los Angeles, Los Angeles, CA 90095, USA; psaggese@mednet.ucla.edu (P.S.); cesaramartinez@mednet.ucla.edu (C.A.M.); 2 Laboratory of Molecular Medicine and Genomics, Department of Medicine, Surgery and Dentistry ‘Scuola Medica Salernitana’, University of Salerno, 84081 Baronissi (SA), Italy; assellitto@unisa.it (A.S.); ggiurato@unisa.it (G.G.); gnassa@unisa.it (G.N.); frizzo@unisa.it (F.R.); rtarallo@unisa.it (R.T.)

**Keywords:** cancer metabolism, mitochondrial metabolism, cancer epigenetics, cell differentiation in cancer

## Abstract

**Simple Summary:**

Cancer cells change their metabolism to support a chaotic and uncontrolled growth. In addition to meeting the metabolic needs of the cell, these changes in metabolism also affect the patterns of gene activation, changing the identity of cancer cells. As a consequence, cancer cells become more aggressive and more resistant to treatments. In this article, we present a review of the literature on the interactions between metabolism and cell identity, and we explore the mechanisms by which metabolic changes affect gene regulation. This is important because recent therapies under active investigation target both metabolism and gene regulation. The interactions of these new therapies with existing chemotherapies are not known and need to be investigated.

**Abstract:**

Metabolic reprogramming is a hallmark of cancer, with consistent rewiring of glucose, glutamine, and mitochondrial metabolism. While these metabolic alterations are adequate to meet the metabolic needs of cell growth and proliferation, the changes in critical metabolites have also consequences for the regulation of the cell differentiation state. Cancer evolution is characterized by progression towards a poorly differentiated, stem-like phenotype, and epigenetic modulation of the chromatin structure is an important prerequisite for the maintenance of an undifferentiated state by repression of lineage-specific genes. Epigenetic modifiers depend on intermediates of cellular metabolism both as substrates and as co-factors. Therefore, the metabolic reprogramming that occurs in cancer likely plays an important role in the process of the de-differentiation characteristic of the neoplastic process. Here, we review the epigenetic consequences of metabolic reprogramming in cancer, with particular focus on the role of mitochondrial intermediates and hypoxia in the regulation of cellular de-differentiation. We also discuss therapeutic implications.

## 1. Introduction

Metabolic reprogramming is a defining characteristic of cancer. Otto Warburg originally observed an increased dependency of cancer cells on glycolysis even in the presence of oxygen, now defined as the Warburg Effect [1]. Based on his observation, Warburg hypothesized that cancer is caused by defects in mitochondrial metabolism. However, later studies have shown that, even if mitochondrial metabolism can be altered in cancer cells, mitochondria are still functional in most cancers, and play a significant role in cancer development and progression [2,3]. Indeed, in addition to increased glycolysis, cancer cells are characterized by an increased dependency on glutamine as an anaplerotic metabolite that sustains the mitochondrial tricarboxylic acid (TCA) cycle for energetic and anabolic purposes [2]. Glucose and glutamine are the most abundant metabolites present in serum and in cell culture medium, thus representing one of the main sources of energy necessary for the regulation of several biochemical processes in mammals. The crosstalk between these two pathways and their reprogramming in tumors is well reported in the literature. Both metabolites replenish the tricarboxylic acid cycle [4], contributing to energy production and generation of key intermediates.

The switch from aerobic to glycolytic metabolism of glucose serves two main functions: to provide rapid energy in the form of adenosine triphosphate (ATP) and to shuttle glucose into various biosynthetic pathways necessary for cellular division and redox balance [5]. The ATP yield from glycolysis, while not as efficient as mitochondrial respiration, is produced at a faster rate. In cancer cells, the final end product of glycolysis, pyruvate, is reduced to lactate, restoring the oxidized nicotinamide adenine dinucleotide (NAD+) necessary to sustain glycolysis. This allows for a build-up of intermediates that can feed into anabolic and redox pathways, including the pentose phosphate shunt, serine and hexosamine biosynthetic pathways, and lipid biosynthesis [6]. This results in the rapid generation of the biomass and energy required for the increased proliferative capabilities of cancer cells.

Increased glutamine metabolism also serves bioenergetic and anabolic purposes in cancer cells. Glutamine is converted to glutamate via glutaminolysis by the enzyme glutaminase; glutamate is then converted to the TCA intermediate alpha-ketoglutarate (αKG) by either glutamate dehydrogenase or transaminases. In cancer cells, glutamine-derived αKG can feed the TCA cycle in the canonical direction, with the production of NADH that feeds the electron transport chain and ATP production, or can be channeled in the reverse direction with the production of citrate, which is exported by the mitochondria and used for anabolic purposes [7,8].

Overall, the reasons for metabolic dysregulation in cancer are multifaceted, and are caused by a complex interaction of oncogenic alterations and consequent aberrations in cellular signaling with changes in the tumor microenvironment due to hypoxia and shifts in nutrient availability. The microenvironmental landscape is a major driver of intra-tumoral heterogeneity, which affects tumor progression and response to current and experimental therapies, as ischemic and hypoxic regions within tumors tend to be more prone to drive disease progression, invade surrounding tissues, and escape from therapies [9,10].

In this context, the mitochondria are a central hub of metabolic signaling, which sense the oxygen and nutrient levels and modulate their activity in response to oncogenic and microenvironmental cues. Besides the obvious bioenergetics role, mitochondria are responsible for the regulation of apoptotic cell death, calcium homeostasis, signal transduction, and recent studies have highlighted their role as signaling organelles responsible for modulation of cellular functions through metabolic retrograde signaling to the nucleus [11]. Several metabolites exported by the mitochondria act as substrates or co-factors for nuclear enzymes responsible for epigenetic modification of DNA and histones, affecting gene expression and modulating cell function and differentiation state [12].

Here, we review the impact of metabolic dysregulation in epigenetic-driven cancer progression and de-differentiation, with particular focus on the role of mitochondrial metabolites in the epigenetic regulation of cell differentiation. First, we present an overview of epigenetic regulation in cancer (Section 2), then we explain the role of mitochondrial metabolism (Section 3) and hypoxia (Section 4) in the epigenetic regulation of cell differentiation. We conclude with therapeutic implications (Section 5).

## 2. Epigenetic Regulation in Cancer

During the life cycle of a cell, genetic and epigenetic mechanisms work in concert, modulating gene expression occurring in physiological processes of cell development and proliferation, energy production, and metabolic reactions. In the context of a highly synchronized regulatory mechanism, indeed, the epigenome guarantees that all the cells of the body, despite having the same genome, can differentiate phenotypically into many cell types and tissues that make up the architecture of the organism and allow its functions [13,14].

The epigenome of a cell is a dynamic and flexible entity, able to integrate the genetic information encoded by the DNA sequence with intra- and extra-cellular signals, including cell–cell contact, stimulation by cytokines, hormones, growth factors, and metabolic signals. In multicellular organisms, cell type identity, cell reprogramming and tissue organization are maintained by the epigenome, inherited through multiple cell divisions and extended through a specific lineage [13,14]. Differently from genetic changes, however, epigenetic modifications do not affect DNA sequence, but rather adapt the chromatin structure through covalent modifications that affect DNA bases, histone protein tails, nucleosome position, all together controlling DNA packaging and chromatin accessibility to the transcriptional machinery [15].

The epigenome is influenced by developmental and environmental stimuli to which the organism is exposed such as diet, social interactions, circadian rhythms, aging, exposure to chemicals, infections, therapeutic drugs, psychological state, microbiome, and more, making each individual bear a unique epigenetic and genetic profile that modulates the response to the environment [13,14].

In recent years, epigenome profiling, combined with other genome-wide technologies, has questioned our knowledge of the epigenomic landscape, providing a portrait of epigenetic modifications at a base-pair resolution [16]. Large-scale projects have been conducted by several national and international organizations such as the US National Institutes of Health Roadmap Epigenomics Mapping Consortium [17], the International Human Epigenome Consortium [18], The Cancer Genome Atlas Network [19], BLUEPRINT [20] and the International Cancer Genome Consortium, leading to the creation of a distribution map of epigenetic marks in hundreds of fetal and adult normal and cancerous tissues. Thanks to these efforts it has then become clear that epigenetic dysregulation is a distinctive feature of virtually all human cancers [21].

Indeed, epigenome analyses, combined with the genome sequencing of thousands of different tumors, elucidated a profound disruption of the epigenome in cancer cells, revealing mutations in key genes encoding for the major classes of epigenome regulatory proteins, including DNA methylation, histone proteins and histone modification enzymes, histone mark readers, chromatin-remodeling complexes, as well as proteins that regulate the function of metabolic enzymes. Many of these alterations are involved in tumor initiation and in the main hallmarks of cancer, including cell growth, immune evasion, metastasis and drug resistance, thus demonstrating a causative role for an altered epigenome in cancer [22]. Metabolic reprogramming driven by the oncogenic transformation modulates the activity of histone and DNA modifying enzymes, which in turn regulates the expression of metabolism-associated genes, leading to an interplay between metabolism and epigenetic during carcinogenesis and cancer progression [23].

### 2.1. DNA Methylation

**Figure 1:**
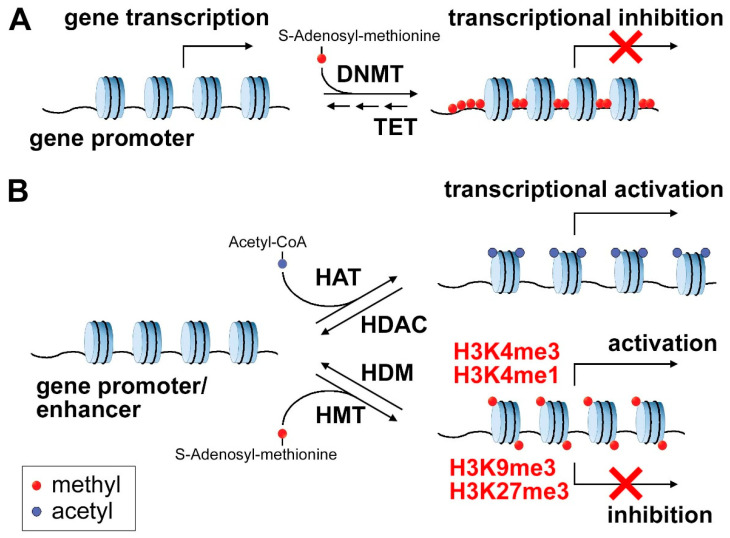
Epigenetic regulation of gene expression. (**A**) DNA methylation on gene promoters causes transcriptional inhibition through compaction of chromatin. (**B**) Histone modifications can occur on gene promoters or enhancers and can cause gene activation or repression: acetylation is associated with gene activation, methylation can be associated with either activation or repression according to the methylated residue, as indicated.

DNA methylation is one of the best-characterized epigenetic marks; in mammals, it occurs mostly on the fifth position of cytosine (5mC). Cytosine methylation is involved in crucial biological processes such as regulation of gene expression during development and differentiation, cell identity specification, transposon silencing, genomic imprinting, and X-chromosome inactivation. DNA methylation is generally restricted to CpG sites most frequently located in the promoters of genes, representing regions of high CG density called CpG islands (CGIs), of 0.5–2 kb in length. The deposition of the methyl marks in CGIs induces chromatin condensation, thus preventing transcription and therefore gene expression; conversely, demethylation is generally associated with the presence of a more relaxed chromatin structure that facilitates transcription (Figure 1A) [24,25]. The human genome contains ~30,000 CGIs, predominantly located within the promoter of housekeeping genes, where DNA remains frequently unmethylated and transcriptionally active, as well as in the promoter of tissue-specific and developmental regulator genes. DNA methylation pattern is established (de novo DNA methylation) during embryonic development through the action of DNA methyltransferase (DNMTs) enzymes DNMT3A and DNMT3B, dynamically regulated by DNMT3C and maintained through multiple cell divisions by a DNMT1-mediated copying mechanism, thus providing a mechanism of epigenetic inheritance [26,27]. DNA methylation is reversed by the activity of ten-eleven translocation (TET) proteins, a class of 5-methyl-cytosine dioxygenases that catalyse hydroxylation of 5mC, the first step of DNA demethylation [28].

The deposition of methyl marks within gene promoter regions determines a direct inhibition of gene expression; however, the silencing mechanism can be also indirect and involve methyl-CpG binding domain proteins that facilitate transcriptional repression by recruiting histone-modifying enzymes and chromatin remodeling complexes [27].

Cancer cells present an abnormal DNA methylation pattern, characterized by two main alterations: genome-wide DNA hypomethylation and local DNA hypermethylation [29] at gene promoters. DNA hypomethylation is generally caused by loss-of-function mutations in DNMT enzymes, while DNA hypermethylation is linked to the up-regulation of DNMTs or to loss-of-function mutations in TET genes [14].

A global loss of DNA methylation promotes the mobilization of transposable elements and genome instability and is associated with oncogenic transformation through the overexpression of proto-oncogenes and growth factors [30]. Germline mutations within DNMT3B gene cause chromosome instability linked to immunodeficiency-centromeric instability-facial anomalies syndrome, a peculiar DNA methylation deficiency disease associated with the development of many cancers [31]. Among growth factors affected by the loss of DNA methylation, the insulin-like growth factor 2 (IGF-2) plays an important role. IGF-2 has a monoallelic expression in normal cells, but a loss of imprinting, due to demethylation of the second allele, leads to uncontrolled proliferation in malignant cells [32].

On the other hand, site-specific promoter hypermethylation is tumorigenic when resulting in the inactivation of genes controlling cell cycle checkpoints and tumor-suppressor genes [33]. Indeed, genes commonly associated with cell cycle regulation and DNA repair such as RB, BRCA1/2 and PTEN are frequently hypermethylated in cancer [34]. DNA hypermethylation has also been associated with DNA damage in microsatellite repetitions in colorectal and ovarian cancers [35], where hypermethylation at the promoter of the DNA-repair gene MLH1 leads to microsatellite instability [36]. The expression of metabolic genes is frequently modulated by DNA methylation in cancer, by directly regulating their transcription or via dysregulation of key signaling pathways [37]. For example, BRCA1 and MLH1 hypermethylation have been associated with increased glycolysis and tumor progression [23]. In breast, liver, gastric and colorectal cancer, fructose-1,6-bisphosphatase 1 and fructose-1,6-bisphosphatase 2, the rate limiting enzymes for gluconeogenesis, are frequently down-regulated via promoter hyper-methylation, thus promoting glycolysis and ATP production to support cancer growth. Glycolysis is also enhanced in hepatocellular carcinoma and glioblastoma through the promoter hypomethylation of the hexokinase 2 enzyme [38]. Signaling cascades that can be influenced by differential DNA methylation contributing to the glycolysis include PI3K/AKT/mTOR and HIF-1 pathways, whose alterations are often sustained by the epigenetic silencing of multiple tumor suppressor genes. Another locus frequently silenced in cancer as a result of promoter hypermethylation is the INK4 locus, encoding for a family of cyclin-dependent kinase inhibitors, such as p14, p15 and p16, representing early indicators of malignant transformation [39]. p16 hypermethylation has a poor prognostic value in non-small cell lung cancer and colorectal cancer [40]. Similarly, the p15 locus is often hypermethylated in hematological malignancies [41].

Mutations of DNMT enzymes also play a critical role in tumorigenesis. DNMT1 alterations have been described in colorectal cancer [42], whereas DNMT3A mutations have been often reported in myelodysplastic syndromes (MDS) and are associated with poor survival in acute myeloid leukemia (AML) [43,44].

Mutations in proteins that erase DNA methylation, including TET2, have been detected in MDS and in myeloproliferative neoplasms [45]. In breast cancer, silencing of the DNA demethylase TET2 causes a defect in luminal differentiation and tamoxifen resistance [46]. In addition, increasing evidence demonstrates that DNA methylation also regulates the expression of non-coding RNAs with pleiotropic activities on gene expression, some of which are also involved in malignant transformation and cancer progression [47].

### 2.2. Histone Marks

Chromatin architecture is dynamically regulated by histone modifier enzymes that covalently modify the N-tails of histone proteins constituting the nucleosome by acetylation, methylation, phosphorylation, ubiquitination and sumoylation, thus regulating DNA accessibility and gene expression. Histone modifications are regulated by histone acetyltransferases (HATs) and deacetylases (HDACs) that add and remove acetyl groups or by histone methyltransferases (HMTs) and demethylases (HDMs) that introduce and remove methyl groups (Figure 1B).

Transcriptionally active promoters are generally enriched in trimethylated H3 at lysine 4 (H3K4me3), while inactive promoters are characterized by trimethylated H3 at lysine 27 (H3K27me3) or trimethylated H3 at lysine 9 (H3K9me3) marks. Regulatory enhancers are enriched in monomethylated H3 at lysine 4 (H3K4me1) and/or acetylated H3 at lysine 27 (H3K27ac) [48,49]. Alterations in genes encoding histone modification enzymes contribute to the development and progression of human cancers by inducing global changes in the histone modification pattern which may affect the recruitment of the transcription machinery thus contributing to aberrant gene expression [15]. Mutations in HAT genes are frequent in colon, uterine, lung tumors and in leukemia [50] and lead to abnormal acetylation. In hematological cancers, HATs are often involved in chromosomal translocation [51]. Hypoacetylation of the p21waf1/cip1 (CDKN1A) promoter by HDACs is associated with transcriptional silencing of this tumor-suppressor gene, thus contributing to malignant transformation [52]. Histone methylation at lysine residues also plays a crucial role in tumorigenesis. In AML, the tumor suppressor gene p15 present at the INK4B locus is silenced through the deposition of H3K9me marks mediated by the methyltransferase SUV39H1 [53]. Genes associated with mixed-lineage leukemia (MLL) are aberrantly activated through H3K79 methylation induced by DOT1L, recruited in turn by MLL fusion proteins like AF9 and AF10 [54]. Another important protein, SMYD2, plays a critical role in esophageal squamous cell carcinoma and in pancreatic cancer, promoting cell growth by catalyzing H3K36 methylation [55].

In addition, emerging evidence indicates that histone modifications not only regulate chromatin structure and gene expression but also other nuclear processes such as DNA repair [50]. Histone phosphorylation activates proto-oncogenes that drive malignant transformation, such as c-fos and c-jun involved in cell cycle progression and proliferation [56], while polyubiquitination of H2A and H2AX has been associated with aberrations in DNA damage repair machinery [57].

Finally, a novel epigenetic modification, the histone GlcNAcylation, has been described. The N-acetylglucosamine (GlcNAc) is a metabolite produced through the hexosamine biosynthetic pathway, whose up-regulation in cancer cells is mainly driven by an increased glucose uptake. Although the transcriptional implication of the histone GlcNAcylation requires further investigation, this modification seems to be implicated in the glucose and glutamine metabolism and is aberrantly detected in cancer, where it is associated with an increased malignant behavior [58].

### 2.3. Chromatin Remodeling

A critical role in coordinating chromatin accessibility and gene expression is played by specific protein complexes, which can modulate nucleosome repositioning using the energy of hydrolysis of ATP. Among them, one of the best characterized is the multiprotein SWI/SNF chromatin-remodeling complex, mainly implicated in DNA damage response (DDR) and in facilitating double-strand break repair, thus protecting the genome from DNA damage-induced instability and tumorigenesis. The SWI/SNF complex also controls the pluripotency of embryonic stem cells, cell differentiation, and cell cycle.

A large portion of human cancers is characterized by mutations in genes encoding SWI/SNF subunits; furthermore, some oncogenes like MYC and KRAS can interact with SWI/SNF subunits promoting cancer progression in leukemia, lung and colon cancer cells. Inactivation of the SWI/SNF complex results in uncontrolled cell cycle progression and tumorigenesis [59].

Interestingly, many chromatin-modifying enzymes are regulated by substrates or cofactors that are intermediates of cell metabolism, whose concentrations can modulate the activity of the enzymes, thus influencing the chromatin state and linking metabolic perturbation to a dysregulated cell proliferation. For example, phosphoglycerate dehydrogenase (PHGDH) is frequently amplified in melanoma and breast cancer; the PHGDH enzyme is critical for serine biosynthesis and regulates the methyl donor methionine thus modulating the cell methylome and epigenetic state [23]. Finally, increasing evidence demonstrates that chromatin structure and accessibility are not only regulated by modifications of DNA and histones but also by non-coding RNAs. Small RNAs with oncogenic potential [60] and long non-coding RNAs can play a critical function in nucleosome positioning by binding to DNA and chromatin remodelling complexes [61]. miRNAs, for example, can function as oncogenes or tumor suppressors in several cellular pathways, including glutaminolysis, glycolysis, and TCA cycle [23].

## 3. Metabolic Dysregulation as a Driver of Cell Fate in Stem Cells and Cancer

In addition to its importance in meeting the bioenergetic and anabolic demands of cancer cells, metabolic dysregulation also affects cell signalling regulating key cellular biological processes, including gene expression and cellular differentiation [62].

**Figure 2:**
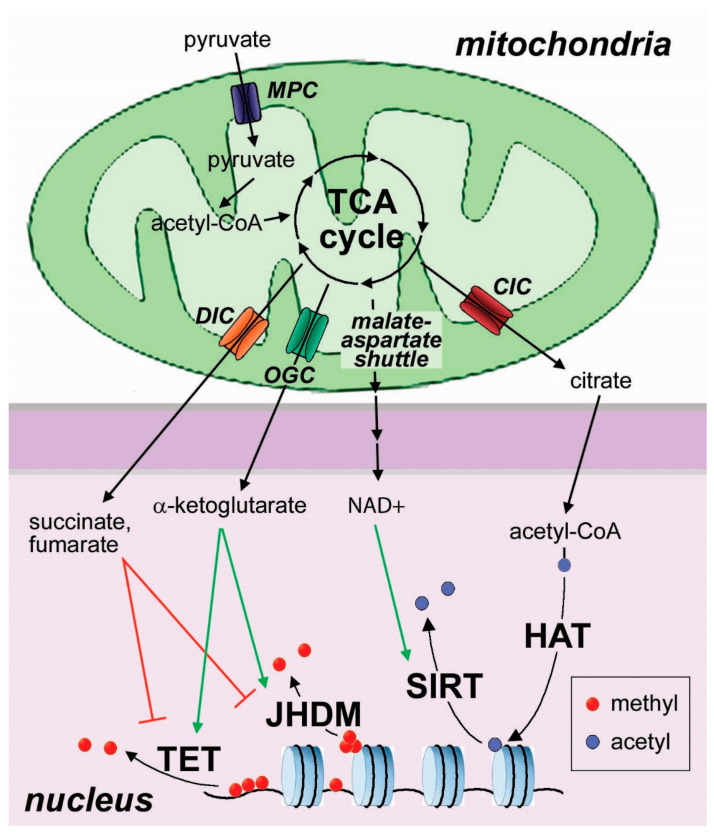
Mitochondrial signaling to the nucleus. The mitochondria act as a metabolic signaling hub, sensing the bioenergetic and oxidative status of the cell and sending signals to the nucleus to regulate gene expression. The mitochondrial pyruvate carrier (MPC), by providing pyruvate to replenish the TCA cycle, is essential for stem cell differentiation. Metabolites exported by the mitochondria through the mitochondrial transport system regulate nuclear enzymatic activities, resulting in epigenetic changes. αKG, exported by the αKG carrier (OGC), activates Jumonji domain-containing histone demethylases (JHDM) and DNA hydroxylases ten-eleven translocation (TET). Succinate and fumarate, exported by the dicarboxylate transporter (DIC), compete with αKG for binding to these enzymes. Oxidized NAD, exported via the malate-aspartate shuttle, activates the histone deacetylases of the sirtuin family. Acetyl-CoA can derive from citrate exported by the mitochondria through the citrate carrier (CIC), and is required as a substrate for histone acetylation.

The link between metabolism and epigenetics is mainly due to the fact that many chromatin-modifying enzymes require a specific metabolite to function [63,64]: acetyl-CoA provides the substrate for histone acetylation; methyl-transferases require s-adenosyl-methionine as methyl donor, relying on one-carbon metabolism; NAD+ is required for the activity of a class of histone deacetylases, the sirtuins [65]; several demethylation reactions use α-ketoglutarate (αKG) as a co-factor to remove methyl groups from histones and DNA [66]. αKG is required for the activity of some Jumonji-domain-containing histone demethylases (JHDM) and the TET enzymes, which initiate DNA demethylation (Figure 2) [67]. These enzymes also use other co-factors, such as ferrous iron, oxygen, and ascorbic acid. As a consequence, a hypoxic microenvironment can modulate epigenetic regulation of cell differentiation, as we will discuss in Section 4. Ascorbic acid is also required for TET-dependent thymocyte maturation, and its deficiency has been associated with the accumulation of undifferentiated hematopoietic stem cells and leukemogenesis [68]. Moreover, competitive inhibitors with structural similarity to αKG such as the TCA intermediates succinate and fumarate, and the oncometabolite 2-hydroxyglutarate in its enantiomeric forms D- and L-2-hydroxyglutarate, can interfere with the activity of αKG-dependent demethylases [69]. As a consequence, the activity of DNA and histone demethylases is modulated by the balance of αKG and its competitor metabolites (fumarate, succinate, 2-hydroxyglutarate), as well as the availability of other co-factors including oxygen and ascorbic acid (Figure 2).

### 3.1. Mitochondrial Metabolism in the Regulation of Cell Differentiation in Embryonic Stem Cells

Several studies have reported the critical role of mitochondrial metabolites in the regulation of cellular fate. This effect is linked to the availability of intermediates that function as substrates or co-factors of enzymes responsible for chemical modifications of DNA, RNA, and histones [70,71], required to establish specific epigenetic patterns and gene expression programs that regulate the balance between self-renewal and lineage differentiation [72].

In embryonic stem cells, αKG and NAD+ are major regulators of differentiation. αKG is required for maintenance of pluripotency and its levels drop during differentiation, causing a global increase in H3K9me3 and H3K36me3 marks [73]. Production of αKG from glucose is essential to prevent differentiation in embryonic stem cells, concomitantly with DNA demethylation of germline-associated, TET-activated genes, and reduction in H3K27me3 on specific gene promoters; H3K27me3 is demethylated by αKG-dependent UTX and JMJD3 demehtylases [67]. TET-mediated DNA de-methylation is required to activate pluripotency transcription factors (NANOG, SOX2, OCT4 and KLF4) target genes and maintain pluripotency in embryonic stem cells [74]. Consistently, ascorbic acid is also required to maintain DNA hypomethylated and preserve pluripotency in embryonic stem cells [75]. Conversely, recruitment of the polycomb repressor complex, which methylates H3K27, is essential to repress differentiation-related genes in embryonic stem cells [76,77], and αKG induces multilineage differentiation of human pluripotent stem cells [78]. Thus, paradoxically both αKG-dependent histone demethylation and polycomb-dependent histone methylation are required for maintenance of the pluripotent state in embryonic stem cells. The mechanisms that target the changes in histone marks on specific genes in response to global fluctuations of αKG are currently unknown.

NAD+-dependent sirtuins are important epigenetic regulators determining embryonic stem cell phenotype. SIRT1 directly represses differentiation-related genes by de-acetylation [79]. SIRT1 deacetylases and represses DNMT3I in embryonic stem cells, thus reducing the methylation and repression of germline-specific genes and maintaining the differentiation potential [80]. SIRT1 is also required for myc-dependent activation of methionine adenosyltransferase 2a, which converts methionine to S-adenosyl-methionine and provides the substrate for H3K4 tri-methylation required for pluripotency [81]. Conversely, SIRT6 is required to suppress pluripotency genes OCT4, SOX2 and NANOG during embryonic differentiation by deacetylation of H3K56 [82]. Similarly, SIRT2 is required for metabolic reprogramming during embryonic stem cell differentiation; however, this effect seems to be mediated by direct deacetylation of glycolytic enzymes, and not by epigenetic mechanisms [83]. Therefore, the effect of NAD+ levels on cell differentiation depends on the cellular context, the pattern of sirtuins expressed in the cell, and the combination of epigenetic and non-epigenetic effects of protein deacetylation mediated by these enzymes.

### 3.2. Mitochondrial Metabolism in the Regulation of Cell Differentiation in Cancer Cells

In cancer cells, lineage plasticity can manifest in different ways, including cancer stem cell phenotype, epithelial-to-mesenchymal transition (EMT), and trans-differentiation. Cancer stem cells are considered to be resistant to chemotherapy because of increased drug efflux, enhanced protection against reactive oxygen species, and the ability to adopt a quiescent state [84,85]. EMT is associated with the development of a migratory and stem-like phenotype, associated with metastasis and drug resistance [86]. Finally, cancer cells can escape treatments by trans-differentiating to a different cell lineage, including the transition to a neuroendocrine-like, small-cell phenotype [87]. These cell transitions require extensive chromatin remodeling and are susceptible to metabolic regulation. The central role of mitochondrial metabolism in the regulation of cell differentiation has two important consequences. First, nuclear or mitochondrial DNA mutations that impair mitochondrial enzymatic functions can accelerate carcinogenesis and cancer de-differentiation. Second, alterations in mitochondrial function due to nutrient deprivation in the tumor micro-environment can drive cellular de-differentiation and drug resistance.

Alterations in mitochondrial enzymes can cause aberrant methylation of histone tails; for example, mutations of isocitrate dehydrogenase 1 (IDH1) and 2 (IDH2), the enzymes responsible for the production of αKG from isocitrate, induce DNA hypermethylation and impaired differentiation in hematopoietic cells [88]. Indeed, in gliomas and leukemia alterations in the same enzymes lead to the accumulation of an oncometabolite, 2-hydroxyglutarate (2-HG), which inhibits HDMs and TET proteins by competing with αKG. In addition, the inhibition of demethylases promotes tumorigenesis by blocking cell differentiation and activating the mammalian target of rapamycin (mTOR) pathway [89]. Mutations in fumarate hydratase (FH) and succinate dehydrogenase (SDH) cause accumulation of succinate and fumarate, and consequent inhibition of αKG-dependent demethylases. This can lead to pathogenic differentiation programs with potential implications for cancer cells [90] and EMT [24]. P53 reactivation in p53-null pancreatic ductal adenocarcinoma cells has been shown to increase the glucose-dependent production of αKG and induce cell differentiation concomitant with DNA demethylation [91].

Mutations in mitochondrial DNA (mtDNA) and changes in mtDNA copy number have been involved in cancer progression and chemoresistance [92]. The precise role of mtDNA mutations has not been studied in detail, mostly for the absence of experimental models [93]. It has been observed that cancer stem cells have reduced mtDNA copy number, consistently with their increased glycolytic phenotype [94]. This change in copy number mirrors physiological changes that occur during development: embryonic stem cells have low mtDNA copy number, and expand their mtDNA upon differentiation [95]. Interestingly, reduction of mtDNA copy number is associated with increased genomic DNA methylation, possibly due to reduced activity of αKG-dependent demethylases [96].

Even in the absence of mutations in genes involved in mitochondrial metabolism, the rapid and chaotic proliferation of tumor cells often leads the cancerous tissue to outgrow its vascular bed. The tumor microenvironment is thus characterized by steep gradients of nutrient and oxygen concentration, which affect regional mitochondrial metabolism. For instance, glutamine deprivation in the ischemic core of melanomas and lung cancers causes histone hypermethylation driving tumor de-differentiation [97]. Among the most studied histone modification enzymes, correlated with epigenetic silencing, are the polycomb repressor complexes 1 (PRC1) and 2 (PRC2). Enhancer of zeste 2 (EZH2), a member of PRC2, represents the catalytic subunit involved in histone methyltransferse activity with substrate specificity to H3K27 [98]. H3K27me3 is involved in the silencing of genes responsible for tumor suppression and differentiation. Regional glutamine deficiency in the tumor core leads to depletion of αKG levels, which is a critical cofactor for histone demethylases. The low glutamine levels reduce the activity of JMJD3 and UTX lysine demethylases, leading to histone hypermethylation. Specifically, H3K27me3 represses the expression of melanocyte differentiation genes, such as PMEL, KIT, and GJB1, causing a block of cell differentiation [97]. Glutamine deprivation also induces WNT activation and induces self-renewal and stemness in colorectal epithelial cells haploinsufficient for the tumor suppressor APC. This effect is rescued by supplementation of αKG in vitro and in vivo [99].

NAD+-dependent sirtuins have also been associated with cancer de-differentiation. SIRT1 is over-expressed in brain, breast, colorectal cancer, and leukemia. SIRT1 is required for SOX2 expression in liver cancer stem cells by deacetylation and inhibition of DNMT3B [100], and for EMT induction in breast cancer by deacetylation of H4K16 and H3K9 on the promoter of WNT inhibitors secreted frizzled proteins SFRP1 and SFRP2 and of E-cadherin [101]. Nicotinamide phosphoribosyl-transferase (NAMPT), the rate-limiting enzymes for NAD+ synthesis, induces cancer stem cell phenotype through SIRT1 activation [102]. A NAD+-dependent transcriptional network including the transcription factor E2F2 is required for maintenance of self-renewal and radiation resistance in glioblastoma stem cells, and it is blocked by pharmacological or genetic inhibition of NAMPT [103].

### 3.3. Mitochondrial Transporters in the Epigenetic Regulation of Cell Differentiation

The above-mentioned effects of metabolic alterations on the epigenetic regulation of cell differentiation are mediated by the exchange of metabolites and reducing equivalents between the mitochondria and the nucleus (Figure 2). Whereas the outer mitochondrial membrane is largely permeable to small, negatively charged metabolites thanks to the voltage-dependent anion channel VDAC, the transport of metabolites across the inner mitochondrial membrane is strictly dependent on the activity of a family of selective transporters, overall classified as a mitochondrial transport system. These transporters are responsible for the uptake and export of metabolites by the mitochondria, and therefore mediate the metabolic communication of these organelles with the rest of the cell [4] (Figure 2). Two major components of the mitochondrial transport system have been involved in the epigenetic regulation of cell differentiation in normal stem cells and in cancer: the mitochondrial pyruvate carrier and the αKG-glutamate transporter.

Pyruvate, derived from glycolysis, is imported into mitochondria by the mitochondrial pyruvate carrier (MPC), a hetero-oligomeric protein complex made of two subunits, MPC1 and MPC2. In the mitochondrial matrix, pyruvate is oxidized by the pyruvate dehydrogenase complex generating acetyl-CoA, which then enters the TCA cycle [104]. Pyruvate transport by MPC is a limiting step in this process, and is, therefore, a gatekeeper of fundamental importance in establishing cell metabolic programming [105]. Up-regulation of MPC activity is associated with differentiation of intestinal stem cells into mature enterocytes, and its inhibition is associated with stem cell proliferation, concomitantly with reduced cellular citrate, malate and αKG, and reduced histone acetylation of H3K27 and H3K4 [106]. In this context, αKG reduction in MPC-deficient cells is likely to cause defective histone demethylase activity and histone hypermethylation, leading to cell de-differentiation. Loss of MPC accelerates the glycolytic switch of cancer cells and promotes intestinal tumorigenesis [107]. The overall effect of MPC deletion, however, is context-specific, as blocked pyruvate import in the mitochondria can also cause increased usage of glutamine to replenish the TCA cycle, with consequent depletion of glutathione and cell toxicity in hepatocellular carcinoma [108] and prostate cancer [109].

The αKG carrier (OGC) or SLC25A11 exchanges αKG with malate and can work in either direction. Given the central role of αKG in the epigenetic regulation of cell differentiation, the export of this metabolite from mitochondria likely affects cell differentiation in cancer. Consistently, mutations in OGC that cause reduced export of mitochondrial αKG have been associated with familial paragangliomas, with a hypermethylator phenotype similar to those caused by SDH and FH mutations [110]. In addition to the role in the export of αKG, OGC cooperates with other transporters in shuttling reducing equivalents across the mitochondrial membrane, which can also affect cell differentiation through the regulation of sirtuins.

**Figure 3:**
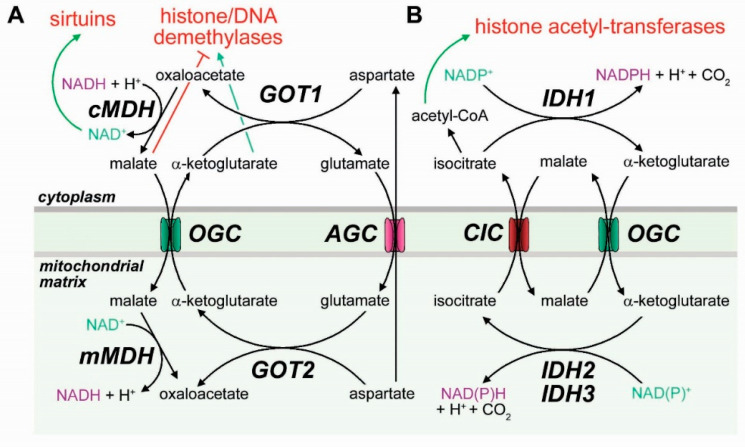
Mitochondrial shuttle systems are essential for the mitochondrial metabolic signaling to the nucleus. (**A**) The malate-aspartate shuttle involves cycling of αKG/malate and glutamate/aspartate between the cytoplasm and the mitochondria, with a net movement of reducing equivalents from the cytoplasm to the mitochondrial matrix in the form of NADH. The increased cytoplasmic NAD+ produced by the net activity of the shuttle can activate the NAD+-dependent deacetylases (HDACs) of the sirtuin family. The exchange of αKG for malate can increase the activity of histone and DNA demethylases. (**B**) The citrate-αKG shuttle involves cycling of citrate/isocitrate, malate, and αKG between the cytoplasm and the mitochondria, with a net movement of reducing equivalents from the mitochondrial matrix to the cytoplasm in the form of NADPH. The isocitrate exported by the CIC can also be converted in the cytosol to acetyl-CoA, which acts as a substrate for histone deacetylases. The balance between different mitochondrial export systems determines the metabolic signaling sent to the nucleus in response to metabolic cues.

As part of the malate-aspartate shuttle (Figure 3A), OGC exports mitochondrial αKG in exchange for cytosolic malate. Malate brings in reducing equivalents, released in the mitochondrial matrix by malate dehydrogenase, which converts malate to oxaloacetate. Glutamate-oxaloacetate transaminase and the aspartate-glutamate carrier AGC complete the cycle, resulting in a net import of reducing equivalents in the form of NADH from the cytosol to the nucleus (Figure 3A). A reduced cytoplasmic NAD+ affects the activation of sirtuins, which are major regulators of cell differentiation. It has been suggested that the role of OGC in the malate aspartate shuttle is essential for lung cancer and melanoma development [111].

As part of the citrate-αKG shuttle, OGC imports cytosolic αKG in exchange for mitochondrial malate. In this case, OGC cooperates with citrate carrier to move reducing equivalents out of the mitochondria via citrate, which is converted to αKG by IDH1 (Figure 3B). This process is essential for biosynthetic and antioxidant processes [112,113]. Therefore, OGC is at the center of a delicate balance, and the direction of its activity is likely modulated predominantly by the energetic and redox status of the cell, in addition to the relative gradients of several metabolites (aspartate, citrate, malate, glutamate) across the inner mitochondrial membrane. By regulating the exchange of αKG and reducing equivalents between the mitochondria and the cytoplasm, mitochondrial activity modulates the cell epigenome and regulates differentiation state in response to metabolic stimuli.

## 4. The Role of Hypoxia in Cancer Cell Differentiation

Since mitochondrial activity is strictly connected to an oxygen supply, hypoxia plays a central role in the epigenetic regulation of cell differentiation. The key players in the response to low oxygen levels are hypoxia-inducible factors (HIFs), which regulate the transcription of a set of genes involved in cancer development and progression [114,115], making them important prognostic and diagnostic biomarkers, as well as a potential targets for novel cancer therapies [116,117]. The relationship between hypoxia and stemness is multi-layered involving at least three different and intertwined mechanisms: (1) transcriptional activation of hypoxia-dependent transcriptional programs through HIFs; (2) impaired mitochondrial oxidation with alterations of the pattern of mitochondrial metabolite export; (3) direct effect of low oxygen on enzymatic activities of epigenetic modifying enzymes.

(1) HIF is an α/β heterodimer that binds the hypoxia response elements (HREs) on the promoters of target genes, activating the hypoxic response. Whereas the β subunit is constitutively expressed, HIF-1α and HIF-2α are regulated post-translationally by an O_2_-dependent mechanism through hydroxylation of proline residues by prolyl hydroxylases (PHDs) and subsequent ubiquitination by pVHL [118,119]. The PHD enzymes are dioxygenases that require for their activity oxygen, as well as iron and αKG [120]. Therefore, HIF-1α/HIF-2α are constitutively degraded during normoxia, whereas during hypoxia they escape ubiquitination and proteasomal degradation and heterodimerize with HIF-1β to regulate transcription of downstream genes [121]. HIF target genes are involved in the regulation of cell proliferation, angiogenic signalling and altered metabolism [122]. The impact of HIFs on cancer stem cells is mostly attributed to the HIF-dependent activation of stemness factors. Hypoxia signalling can induce HIF-dependent expression of known pluripotent factors, such as KLF4, MYC, OCT4, SOX2, and NANOG, which have an important role in the dedifferentiation process under hypoxic conditions, inducing cancer stemness and repressing cancer cell differentiation [123]. It has been reported that cancer stem-like cells can be induced through de-differentiation under hypoxic conditions in glioma, hepatoma and lung cancer, providing new evidence about tumor development and chemoradiotherapy resistance [124].
(2) Reduced availability of oxygen for the mitochondrial respiratory chain is expected to slow down the oxidation of NADH in the mitochondria, leading to feedback inhibition of the TCA cycle. Hypoxia has been shown to stimulate stemness of breast cancer cells by the production of reactive oxygen species, and this effect is mediated by attenuation of the TCA cycle and accumulation of fumarate [125]. Although the epigenetic consequences of these alterations have not been investigated in detail, they are likely to play a role in cell de-differentiation induced by hypoxia. Prolonged hypoxia can cause reduced activity of nuclear pyruvate dehydrogenase, resulting in reduced nuclear availability of acetyl-CoA and reduced histone acetylation [126].
(3) An increasing number of studies have focused on the involvement of hypoxia in regulating multiple components of the epigenetic machinery, including DNA methylation, histone modification, non-coding RNAs (ncRNAs) and chromatin remodelling, affecting the expression of specific genes involved in cancer progression, dedifferentiation, and drug resistance [127,128]. Hypoxia interferes with the activity of oxygen-dependent dioxygenases, including the TET 5-methyl-cytosine dioxygenases and Jumonji-C (JMJC) histone demethylases [129]. As a consequence, hypoxia induces increased trimethylation marks on H3K4 and H3K36 in HeLa cells and in a human fibroblast line, and on H3K9 in several cell lines, including colon carcinoma and epithelial human breast cancer cell lines. These events are driven by the inactivation of the JMJC-containing enzymes, such as lysine demethylase 4A (KDM4A) and 5A (KDM5A). The lack of demethylase activity in hypoxic conditions leads to transcriptional repression of target genes and a phenotype of increased tumor aggressiveness, invasion, migration, and proliferation [130,131]. In addition, hypoxia is able to induce specific changes in the chromatin state associated with EMT. Wu et al. reported that under hypoxia, HDAC3 interacts with hypoxia-induced WDR5, recruits the HMT complex to increase the methylation marks on H3K4, and activates the transcription of mesenchymal genes [132]. Interestingly, the activity of lactate dehydrogenase A, under hypoxic conditions, is able to produce the oncometabolite L-2-hydroxyglutarate, independently of IDH. This event promotes the inhibition of KDM4C and enhanced H3K9 methylation, thus altering the expression of genes involved in cellular differentiation [133,134]. Thienpont et al. have also observed that increased promoter methylation is associated with reduced activity of TET enzymes, which decreases 5-hydroxymethyl-cytosine at gene promoters and enhancers, supporting the hypothesis that hypoxia inhibits TET-mediated DNA demethylation in tumors [135].

## 5. Therapeutic Implications

The epigenetic effects of metabolic alterations in cancer have important consequences for therapeutic purposes. There are two considerations that need to be made in this regard. First, the regional metabolic adaptations in the tumor microenvironment can generate particularly resistant cells. The hypoperfused areas of the tumor are inherently more resistant to therapies for two reasons: one is the reduced delivery of therapeutic action, the other is intrinsic resistance of the cells adapting to survival in this niche. Hypoxia is associated with resistance to radiation treatment, which requires the availability of oxygen for the achievement of a therapeutic effect [136]. In hypoperfused regions, drug concentrations are lower than in the blood, making it harder to achieve therapeutic concentrations in these tumor regions. In addition, cancer cells adapted to survive at long distances from blood supply often show particular resistance to the cytotoxic effect of drugs [137,138]. In this context, the cellular de-differentiation associated with restricted nutrient supply and hypoxia contributes to therapy resistance [139]. It has also been proposed that systemic nutritional deficiency of ascorbic acid, by causing reduced TET activity and DNA hypermethylation in hematopoietic stem cells, contributes to leukemogenesis [68]. Consistently, patients with hematologic malignancies have lower body levels of ascorbic acid [140]. Even if oral ascorbic acid has not yielded the expected results against advanced cancers, high-dose intravenous ascorbic acid is under investigation as an epigenetic and pro-oxidant therapy in several cancers [141].

In addition to the nutrient and oxygen restriction imposed on cancer cells by the solid growth of tumors, also therapeutic interventions can interfere with the uptake or metabolism of critical nutrients in an effort to stunt tumor growth. Recently, a renewed interest in metabolism as an emerging hallmark of cancer has spurred research into identifying metabolic vulnerabilities of cancer cells that can be exploited therapeutically. Novel therapies include glucose uptake inhibitors [142,143,144], glutamine inhibitors [145], glycolysis inhibitors [146,147,148], and mitochondrial metabolism inhibitors [149,150]. A comprehensive review of metabolic therapies is beyond the scope of this review. However, we need to note that in addition to the effect of slowing down cellular metabolism and growth, metabolic therapies targeting glucose, glutamine, and mitochondrial metabolism have the potential to induce epigenetic changes triggering reprogramming and de-differentiation, thus expanding the pool of cancer stem cells. Therefore, the epigenetic effects of these novel metabolic therapies will have to be investigated in detail once these therapies are translated to the clinic, as cellular de-differentiation will represent a likely mechanism of resistance to metabolic therapies as well as it is for genotoxic and radiation therapies.

The epigenetic reprogramming leading to cell de-differentiation and cancer resistance to therapies can be modulated by epigenetic therapies. Several new drugs regulating the epigenetic modifications to histones and DNA are under pre-clinical and clinical investigation, including histone deacetylase inhibitors (HDACi), histone methyl-transferase inhibitors (HMTi), and DNA metyl-transferase inhibitors (DNMTi).

The HDACi vorinostat, romidepsin, and belinostat have been approved by the FDA for the treatment of cutaneous and/or peripheral T-cell lymphoma, and panobinostat for multiple myeloma [151]. Many others are undergoing clinical trials for safety and efficacy. HDACi are believed to exert their anti-cancer action by targeting abnormal epigenetic patterns present in cancer cells. They can induce cell cycle arrest, apoptosis, autophagy in cancer cells, inhibit neoangiogenesis, and modulate immune responses [151]. However, the increased histone acetylation caused by HDACi can increase the accessibility of chromatin to transcription factors and facilitate cellular reprogramming to a stem-like cell state [152,153]. Embryonic stem cells show a permissive chromatin configuration with increased histone acetylation concomitant with H3K27me3 mark on some differentiation-related genes [154].

The HMTi tazemetostat inhibits the activity of EZH2, it is the only HMTi approved by the FDA for the treatment of epithelioid sarcoma, and is under clinical investigations for malignant mesothelioma, hematologic malignancies, and advanced solid tumors [155]. Treatment with EZH2 inhibitors is being investigated in follicular and B-cell lymphomas with activating EZH2 mutations [156], and in solid tumors with overexpression of EZH2 [157,158], which are associated with a poorly differentiated phenotype [159]. EZH2 inhibitors are therefore expected to induce cancer cell differentiation and reduced activity of cancer stem cells. EZH2 inhibitor EPZ011989 re-sensitizes metastatic renal cell carcinoma cells to tyrosine kinase inhibitor sunitinib [160]. Other HMTi are under investigation to prevent the development of resistance. Inhibition of H3K9 methyl-transferase G9a has been shown to overcome drug resistance in head and neck and lung cancers [161,162]. Inhibition of DOT1L, which methylates H3K79 activating target genes, effectively blocks the proliferation of antiestrogen-resistant breast cancer cells [163].

HMTi represent a promising new therapeutic strategy for different hematologic and solid tumors. However, the activity of histone methyl-transferases and demethylases is highly context-specific, and in some systems, the opposite enzymes, histone demethylases, have been reported as responsible for the maintenance of cancer stem cell phenotype. For instance, in glioblastoma cells, the demethylases KDM6A/B, which target H3K27, are required for the maintenance of a subpopulation of quiescent cancer stem cells and their pharmacological inhibition with GSKJ4 selectively targets slow-cycling cancer stem cells [85]. GSKJ4 induces glioblastoma cell differentiation and is effective in vivo against chemotherapy-resistant tumors [164]. Pharmacological inhibition of H3K4 demethylase LSD1 relieves a differentiation block in acute myeloid leukemia cells, sensitizing them to retinoic acid-induced differentiation [165].

The DNMTi 5-azacitidine and decitabine are FDA approved for the treatment of myelodysplastic syndrome and are under investigation for several cancers [166]. They also act as DNA synthesis inhibitors. 5-azacitidine can sensitize gastric cancer cells to chemotherapy by de-repressing miR-129-5p and inhibiting multi-drug resistance transporters [167]. Treatment with 5-azacitidine also sensitizes lung cancer cells to chemotherapy by re-activating the WNT repressor SFRP1 [168].

Recently, a combination of different epigenetic drugs has shown promising results in reversing drug resistance. The combination of EZH2 inhibitor 3-deazaneplanocin A and HDACi panobinostat sensitizes resistant glioblastoma cells to temozolomide [169]. The combination of EZH2 inhibitor EPZ-6438 and DNMTi 5-azacytidine can sensitize multiple myeloma cells to thalidomide [170]. DOT1L inhibitor EPZ-5676 synergizes with DNA hypomethylatig agents in sensitizing leukemia cells to chemotherapy [171]. The combination of DNMTi decitabine and HDACi belinostat resensitizes resistant ovarian cancer cells to cisplatin [172].

## 6. Concluding Remarks

The tight and complex relationship between metabolism and epigenetic regulation of cell differentiation has been studied extensively. However, many questions remain unanswered. A major challenge is the identification of the determinants of cellular response to metabolic alterations like glutamine deprivation and fluctuations in αKG levels, which appear to be context-dependent and to have opposite effects on different cell types or cell differentiation states. The elucidation of the targeting mechanisms linking global fluctuations in cell metabolites to specific epigenetic and transcriptional signatures will allow the rational design of more effective combination treatments and the identification of biomarkers to predict response to determinate epigenetic therapies.

## Abbreviations

5mC	5-methyl-cytosine
AGC	aspartate-glutamate carrier
AML	acute myeloid leukemia
ATP	adenosine triphosphate
CGI	CpG island
CIC	citrate carrier
DNA	deoxyribonucleic acid
DNMT	DNA methyl-transferase
DNMTi	DNA metyl-transferase inhibitors
GlcNAc	N-acetylglucosamine
GOT1/2	glutamic-oxaloacetic transaminase
H3K27ac	acetylated histone 3 lysine 27
H3K27me	methylated histone 3 lysine 27
H3K4me	methylated histone 3 lysine 4
H3K9me	methylated histone 3 lysine 9
HAT	histone acetyl-transferase
HDAC	histone deacetylase
HDACi	histone deacetylase inhibitors
HDM	histone demethylase
HIF	hypoxia inducible factors
HMT	histone methyl transferase
HMTi	histone methyl-transferase inhibitors
IDH1-3	isocitrate dehydrogenase
IGF	insulin-like growth factor
JHDM	Jumonji-domain containing histone demethylases
cMDH	cytoplasmic malate dehydrogenase
mMDH	mitochondrial malate dehydrogenase
MDS	myelodysplastic syndromes
MLL	mixed-lineage leukemia
MPC	mitochondrial pyruvate carrier
mtDNA	mitochondrial DNA
NAD+	oxidized nicotinamide adenine dinucleotide
NADH	reduced nicotinamide adenine dinucleotide
NAMPT	nicotinamide phosphoribosyl-transferase
OGC	αKG (oxoglutarate)-glutamate carrier
PHD	prolyl hydroxylase
PHGDH	phosphoglycerate dehydrogenase
RNA	ribonucleic acid
TCA	tricarboxylic acid
TET	ten-eleven translocation
αKG	alpha-ketoglutarate
